# Validation of the Chinese Brief Ageing Perceptions Questionnaire (C‐BAPQ)

**DOI:** 10.1002/alz.085228

**Published:** 2025-01-09

**Authors:** Frank Ho‐yin Lai, Andrew Man‐hong Siu, Ben Chi‐bun Yip, Eddie Yip‐kuen Hai, Julie‐anne Lowe, Stella Kai‐wui Chan, Vivian Wai‐yin Chui, Kathy Ka‐ying Yu

**Affiliations:** ^1^ Mental Health Research Centre, The Hong Kong Polytechnic University, Kowloon Hong Kong; ^2^ The Northumbria University Newcastle, UK United Kingdom; ^3^ Department of Health Sciences, College of Health, Medicine and Life Sciences, The Brunel University, London United Kingdom; ^4^ School of Medical and Health Sciences, Tung Wah College, Kowloon Hong Kong; ^5^ Faculty of Health Sciences and Wellbeing, The Sunderland University, Sunderland United Kingdom; ^6^ Northumbria University, Newcastle United Kingdom; ^7^ Occupational Therapy Department, Wong Tai Sin Hospital, Kowloon Hong Kong; ^8^ Hong Kong Sheng Kung Hui Welfare Council Ltd, Hong Kong Hong Kong; ^9^ Salvation Army Hong Kong & Macau Command, Tai Po Multi‐service Centre for Senior Citizen, Tai Po Hong Kong

## Abstract

**Background:**

Understand individuals’ self‐perception of aging is crucial for promoting a positive aging experience, better health with good quality of life, addressing activities participation, and can help by advocating policies and interventions that support the diverse needs of an aging population. This study aims to examine the validity and reliability of the Chinese version of BAPQ (C‐BAPQ) for the healthy older people by assessing the content validity, test‐retest reliability, and correlational analyses with mental health by Depression, Anxiety, and Stress Scale (DASS‐21), quality of life by the Short Form 36 Health Survey (SF‐36) and activity participation by the Model of Human Occupation Screening Tool (MOHOST). Moreover, to study the factor structure of the Chinese version of BAPQ (C‐BAPQ) by using exploratory factor analysis.

**Method:**

A two‐phase study was conducted. Phase 1 included reviews by a panel of experts and a panel of caregivers to evaluate the clarity, understandability and relevance of C‐BAPQ items. Phase 2 field testing examined test‐retest reliability, internal consistency, factor structure, and correlation with the SF36, DASS‐21, and MOHOST in a sample of Chinese older adults who are community dwellers.

**Result:**

Inter‐rater reliability analysis demonstrated moderate agreement in clarity, understandability, and cultural relevance for the C‐BAPQ. With a group of 120 older adults from snow‐ball sampling, the C‐BAPQ demonstrated a high level of internal consistency, with Cronbach’s alpha of 0.99, F = .005, p = .831. The C‐BAPQ was moderately correlated with mental health (r = ‐ .52, p < .001), health status and quality of life (r = .45, p < .001) and activity participation (r = .54, p < .001). Factor analysis yielded a five‐factor structure of the C‐BAPQ which was consistent with the original version.

**Conclusion:**

The C‐BAPQ demonstrates sensitivity in assessing self‐perception of ageing in older adults. Further longitudinal research utilizing a larger, more representative sample would serve to enhance the psychometric rigor and properties of the C‐BAPQ.

